# Casein kinase 1 is recruited to nuclear speckles by FAM83H and SON

**DOI:** 10.1038/srep34472

**Published:** 2016-09-29

**Authors:** Takahisa Kuga, Hideaki Kume, Jun Adachi, Naoko Kawasaki, Maiko Shimizu, Isamu Hoshino, Hisahiro Matsubara, Youhei Saito, Yuji Nakayama, Takeshi Tomonaga

**Affiliations:** 1 Laboratory of Proteome Research, National Institutes of Biomedical Innovation, Health and Nutrition, Ibaraki, Osaka 567-0085, Japan; 2 Department of Biochemistry & Molecular Biology, Kyoto Pharmaceutical University, Yamashina-ku, Kyoto 607-8414, Japan; 3 Department of Frontier Surgery, Graduate School of Medicine, Chiba University, Chuo-ku, Chiba 260-8670, Japan

## Abstract

In some fibroblasts, casein kinase 1α (CK1α) is localized to nuclear speckles, which are sub-nuclear compartments supplying splicing factors, whereas it is recruited on keratin filaments in colorectal cancer cells such as DLD1 cells. In order to obtain a deeper understanding of why CK1α is localized to these different subcellular sites, we herein elucidated the mechanisms underlying its localization to nuclear speckles. CK1α and FAM83H were localized to nuclear speckles in RKO and WiDr colorectal cancer cells, which do not express simple epithelial keratins, and in DLD1 cells transfected with siRNAs for type I keratins. The localization of FAM83H to nuclear speckles was also detected in colorectal cancer cells with a poorly organized keratin cytoskeleton in colorectal cancer tissues. Using an interactome analysis of FAM83H, we identified SON, a protein present in nuclear speckles, as a scaffold protein to which FAM83H recruits CK1α. This result was supported by the knockdown of FAM83H or SON delocalizing CK1α from nuclear speckles. We also found that CK1δ and ε are localized to nuclear speckles in a FAM83H-dependent manner. These results suggest that CK1 is recruited to nuclear speckles by FAM83H and SON in the absence of an intact keratin cytoskeleton.

Casein kinase 1 (CK1) is a family of serine/threonine protein kinases^1^,^2^. Six CK1 isoforms have been identified (α, δ, ε, and γ1-3) in humans. CK1 is known to be involved in diverse cellular processes including circadian rhythms, Wnt signaling, membrane trafficking, cytoskeleton maintenance, DNA replication, DNA damage responses, RNA metabolism, and parasitic infections^1^,^2^,^3^,^4^,^5^,^6^,^7^,^8^. As expected from its diverse functions, more than 140 CK1 substrates have been reported to date^2^. The complex functions of CK1 have been suggested to be regulated by its subcellular localization and interactions with other proteins; therefore, the proteins that interact with CK1 and regulate its subcellular localization need to be identified.

CK1 is localized to the cytoplasm, centrosomes, microtubules, nucleus, membrane structures, and mitochondria^2^,^9^,^10^,^11^,^12^,^13^,^14^,^15^,^16^,^17^,^18^. Previous studies have also suggested that CK1α is present within nuclear speckles, which act as compartments that supply splicing factors to active transcription sites^19^, in several vertebrate cell lines such as normal rat kidney (NRK), Cos-7, and NIH-3T3 cells^3^,^20^. CK1α has been shown to bind to and phosphorylate regulators of mRNA processing such as SR proteins and hnRNP C1^3^,^21^. The phosphorylation of SR proteins dictates splice-site selection and assembles splicing factors on pre-mRNA^22^,^23^,^24^. The phosphorylation of hnRNP C1 by CK1α inhibits the binding of hnRNP C1 to mRNA^21^. These findings indicate that CK1α is involved in mRNA processing.

We previously reported that CK1α is localized on keratin filaments in colorectal cancer cells such as HCT116 and DLD1 cells, and demonstrated that this subcellular localization of CK1α is regulated by FAM83H^8^. FAM83H was originally identified as a protein that plays an important role in the formation of dental enamel because amelogenesis imperfecta is caused by a mutation in the FAM83H gene that leads to a premature termination codon^25^. We found that the expression of FAM83H was increased in colorectal cancer tissues^8^. The overexpression of FAM83H changes the morphology of keratin filaments into cytoplasmic vesicle-like structures due to the excessive accumulation of CK1α on keratin filaments^8^,^26^. On the other hand, the knockdown of FAM83H has been shown to decrease the association of CK1α with keratin filaments, which results in the thickening of keratin filaments^8^,^26^. FAM83H simultaneously binds to CK1α and keratin proteins, thereby recruiting CK1α to keratin filaments^8^,^26^. FAM83H has the CK1-binding motifs F-X-X-X-F in its N-terminal region^8^,^9^,^26^. These findings suggest that FAM83H regulates the organization of the keratin cytoskeleton by properly recruiting CK1α on keratin filaments.

In our previous study^8^, we immunohistochemically examined colorectal cancer tissues collected from 111 patients using an anti-FAM83H antibody, and noted that staining for FAM83H was preferentially detected in the nucleus in a small subset of colorectal cancer tissues (Fig. 1a); therefore, we hypothesized that FAM83H is present in the nucleus and may recruit CK1α to the nucleus rather than on keratin filaments. In the present study, we examined this hypothesis and showed that CK1α is localized to nuclear speckles by FAM83H-mediated recruitment to SON, a protein present in nuclear speckles, in the absence of an intact keratin cytoskeleton.

## Results

### FAM83H is localized to nuclear speckles in a small subset of colorectal cancer tissues

**Figure 1:**
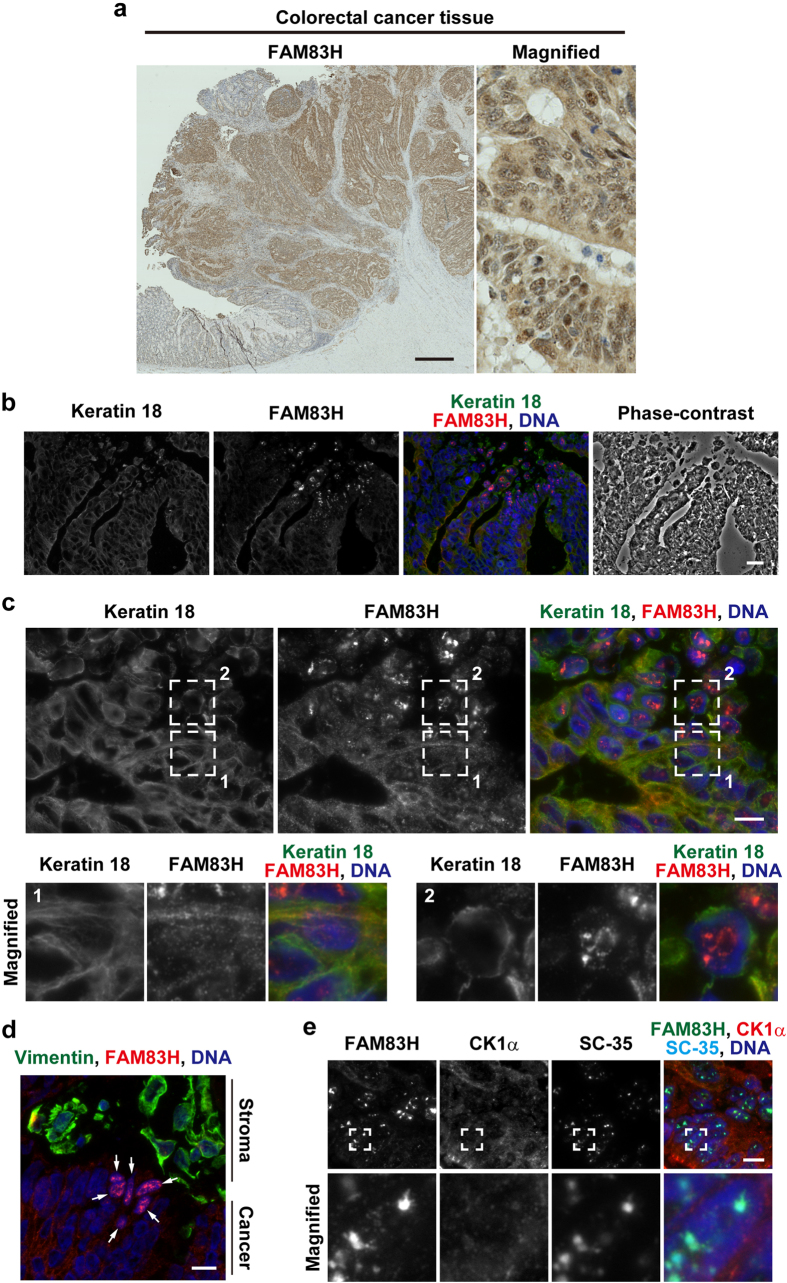
FAM83H is localized to nuclear speckles in colorectal cancer tissues. (**a**) Immunohistochemistry of a paraffin-embedded colorectal cancer tissue sample was performed using an anti-FAM83H antibody (brown). DNA was stained with Mayer’s hematoxylin (blue). (**b–e**) Immunofluorescence staining of a snap frozen colorectal cancer tissue sample was performed using the indicated antibodies and DAPI (for DNA, blue). In (**c,e**) magnified images of the regions enclosed by dotted lines are shown in the margin. In (**d**) arrows indicate cancer cells in which FAM83H was localized to nuclear speckles. Scale bars indicate 1 mm (**a**) 20 μm (**b**) or 10 μm (**c–e**).

We previously stained 111 paraffin-embedded human colorectal cancer tissues using an anti-FAM83H antibody^8^, and detected staining for FAM83H in the nucleus in three of the tissue samples tested (Fig. 1a); therefore, we hypothesized that FAM83H and its interacting protein CK1α may be localized in the nucleus in some colorectal cancer cells.

In order to more clearly detect the nuclear localization of FAM83H in colorectal cancer tissues, we performed an immunofluorescence analysis using snap-frozen colorectal cancer tissues. When 9 colorectal cancer tissues were stained with an anti-FAM83H antibody, the nuclear staining of FAM83H was detected in one tissue sample (Fig. 1b). The nuclear staining of FAM83H was also detected in a small population of cancer cells located at the edge of or detached from the tumor mass. Cancer cells showing the nuclear localization of FAM83H had a poorly organized keratin cytoskeleton (Fig. 1c, region 2), whereas FAM83H was localized on keratin filaments in colorectal cancer cells with rigid and long keratin filaments (Fig. 1c, region 1). Nuclear FAM83H was co-localized with SC-35, which is a marker of nuclear speckles^19^. These results suggest that FAM83H translocates to nuclear speckles from keratin filaments upon the disruption of rigid and long keratin filaments in colorectal cancer cells.

The system of intermediate filaments in cancer cells is known to switch from being keratin-dominant to vimentin-dominant during epithelial-to-mesenchymal transition (EMT)^27^. In order to clarify whether FAM83H translocates to nuclear speckles during EMT, we examined the expression of vimentin in colorectal cancer tissue showing the localization of FAM83H to nuclear speckles. Vimentin was strongly detected in stromal cells, whereas it was absent in cancer cells showing the localization of FAM83H to nuclear speckles (Fig. 1d, arrows). In this colorectal cancer tissue, EMT did not appear to be the cause of the translocation of FAM83H to nuclear speckles.

Although the localization of FAM83H to nuclear speckles was clearly detected in colorectal cancer tissue, we were unable to establish whether CK1α was co-localized with FAM83H in nuclear speckles. We detected the co-localization of CK1α with FAM83H at nuclear speckles in colorectal cancer cells; however, staining for CK1α in nuclear speckles was very weak, and, thus, insufficient for further analyses (Fig. 1e). In order to answer this question, more snap-frozen colorectal cancer tissue samples needed to be analyzed; however, difficulties were associated with the preparation of a large number of these samples.

### CK1α and FAM83H are localized to nuclear speckles in RKO colorectal cancer cells

**Figure 2:**
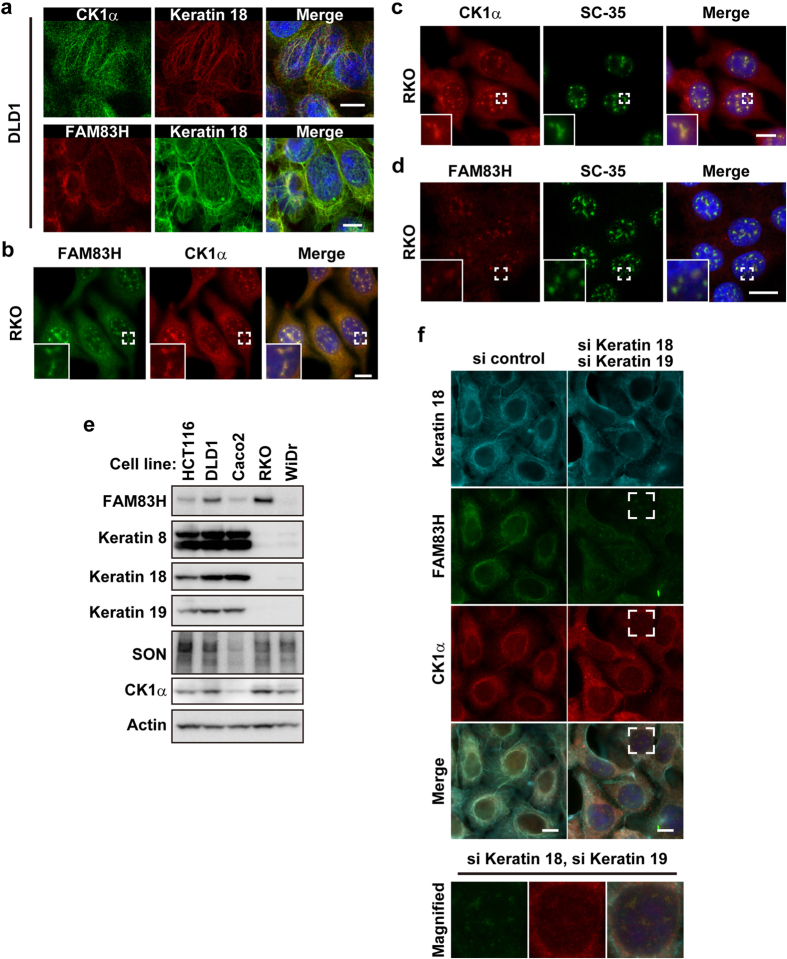
CK1α and FAM83H are localized to nuclear speckles in the absence of an intact keratin cytoskeleton. (**a–d**) DLD1 (**a**) or RKO cells (**b–d**) were subjected to an immunofluorescence analysis. Insets are magnified images at the areas enclosed by white squares. (**e**) A Western blot analysis of colorectal cancer cell lines. (**f**) DLD1 cells were transfected with a combination of siRNAs for keratins 18 and 19 or control siRNA. Cells were analyzed by immunofluorescence. The area enclosed by a white square is magnified in the margin. In (**a–d,f**) DNA was stained with DAPI (blue) and scale bars indicate 10 μm.

In order to perform *in vitro* experiments that reveal the mechanisms by which CK1α is localized to nuclear speckles, we searched for colorectal cancer cell lines in which CK1α is present in nuclear speckles. As shown in our previous study^8^, CK1α and FAM83H were partially co-localized with keratin filaments in DLD1, HCT116, and Caco2 cells (Fig. 2a and data not shown). On the other hand, in RKO cells, CK1α was detected in the nucleus (Fig. 2b,c). CK1α, FAM83H, and SC-35 were co-localized in RKO cells (Fig. 2b–d). These results suggest that CK1α and FAM83H are localized to nuclear speckles in RKO cells.

When RKO cells were immunostained using the anti-keratin 18 antibody, no staining was detected (data not shown); thus, we speculated that RKO cells do not form keratin filaments composed of simple epithelial keratins 8 and 18. In order to assess the expression of simple epithelial keratins in RKO cells, we performed Western blotting using antibodies against keratins 8, 18, and 19. Figure 2e clearly shows that HCT116, DLD1, and Caco2 cells strongly expressed keratins 8, 18, and 19, whereas RKO cells did not. These results support the hypothesis, proposed by *in vivo* experiments, that CK1α and FAM83H are localized to nuclear speckles in colorectal cancer cells without an intact keratin cytoskeleton.

In order to further substantiate the involvement of an intact keratin cytoskeleton in the localization of CK1α and FAM83H to nuclear speckles, DLD1 cells were transfected with siRNA for keratins and analyzed by immunofluorescence using antibodies against CK1α and FAM83H. Since keratin filaments are formed through the specific pairing of type I and II keratins^27^, we expected the depletion of type I keratins 18 and 19 to be sufficient for disrupting the intact keratin cytoskeleton in DLD1 cells. Although transfection with siRNAs for keratins 18 and 19 did not completely deplete these keratins, as judged by immunostaining using the anti-keratin 18 antibody, thick and long keratin filaments were largely diminished (Fig. 2f). As a result, FAM83H and CK1α translocated into nuclear speckles from keratin filaments (Fig. 2f); however, the amount of CK1α entering the nuclear speckles was small (Fig. 2f). Collectively, our *in vivo* and *in vitro* results suggest that CK1α and FAM83H translocate to nuclear speckles when the intact keratin cytoskeleton is disrupted in colorectal cancer cells.

### The localization of CK1α to nuclear speckles depends on FAM83H

**Figure 3:**
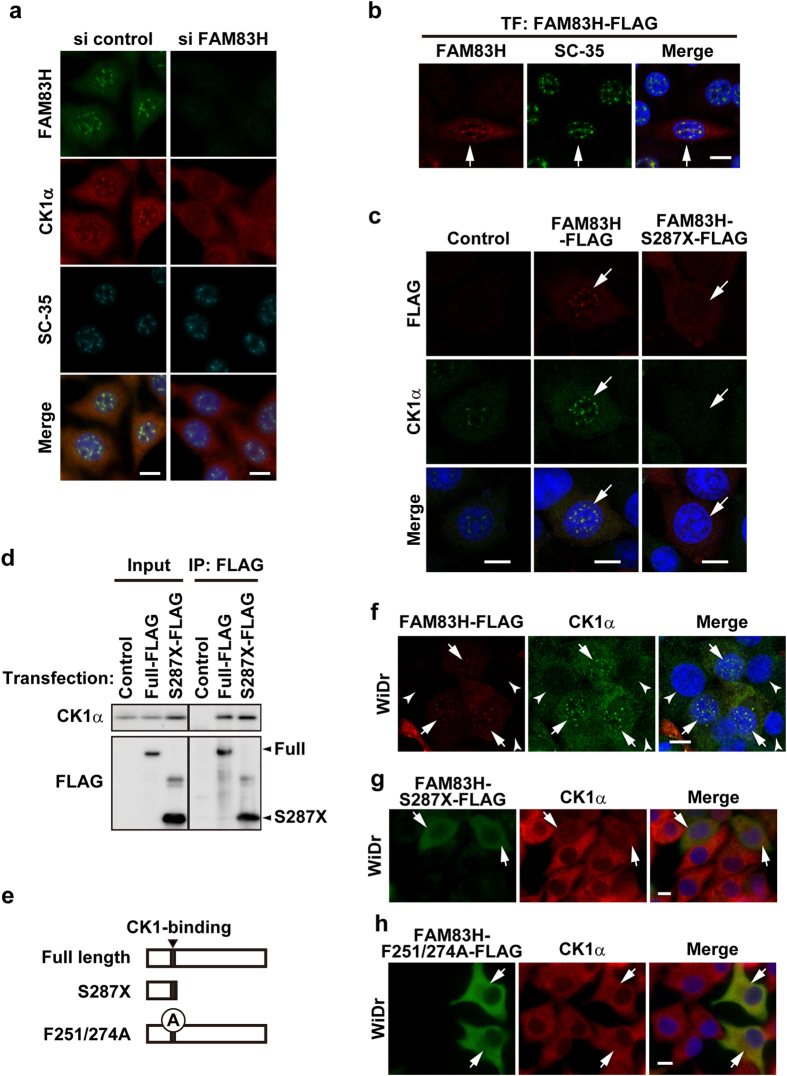
CK1α requires FAM83H to be localized to nuclear speckles. (**a–c,f–h**) RKO cells (**a–c**) or WiDr cells (**f–h**) were transfected with the indicated siRNAs (a) or plasmids encoding the indicated mutants of FAM83H (**b,c,f–h**). Cells were then stained by the indicated antibodies and DAPI (for DNA, blue). Arrows and arrowheads indicate cells expressing and not expressing the proteins encoded in plasmids, respectively. Scale bars, 10 μm. (**d**) Immunoprecipitates using an anti-FLAG antibody and input lysates, which were prepared from RKO cells transiently transfected with plasmids encoding the indicated mutants of FAM83H, were analyzed by a Western blot analysis using an anti-CK1α or anti-FLAG antibody. (**e**) The full length of FAM83H contains 1179 amino acids. FAM83H-S287X is a truncated protein containing 1-286 amino acids and retains the CK1-binding site. FAM83H-F251/274A has alanine substitutions (A) of the two phenylalanines (Phe251 and Phe274) present in the CK1-binding site.

In order to investigate whether the localization of CK1α to nuclear speckles depends on FAM83H, as shown for the localization of CK1α on keratin filaments^8^, we examined the subcellular localization of CK1α in RKO cells depleted of or overexpressing FAM83H. Transfection with siRNA for FAM83H clearly decreased the staining intensity for FAM83H, suggesting the successful depletion of FAM83H (Fig. 3a). In FAM83H-depleted cells, the localization of CK1α, but not SC-35, to nuclear speckles was markedly abolished (Fig. 3a). On the other hand, the overexpression of FAM83H-FLAG, which was localized to nuclear speckles similar to endogenous FAM83H (Fig. 3b), promoted the accumulation of CK1α in nuclear speckles (Fig. 3c). CK1α was clearly co-localized and associated with FAM83H-FLAG, as assessed by immunofluorescence and co-immunoprecipitation assays (Fig. 3c,d), suggesting that CK1α binds to FAM83H in nuclear speckles. RKO cells were further transfected with a dominant negative form of FAM83H, FAM83H-S287X-FLAG (an N-terminal fragment containing CK1-binding motifs^8^) (Fig. 3e). FAM83H-S287X-FLAG bound to CK1α (Fig. 3d) and diminished the localization of CK1α to nuclear speckles (Fig. 3c). These results suggest that CK1α requires FAM83H to be localized to nuclear speckles.

The requirement of FAM83H for localizing CK1α to nuclear speckles was tested further using WiDr cells, which expressed neither FAM83H nor simple epithelial keratins (Fig. 2e). CK1α was not normally detected in the nucleus of WiDr cells (Fig. 3f, arrowheads), whereas its localization to nuclear speckles was clearly induced by transfection with FAM83H-FLAG (Fig. 3f, arrows), but not with FAM83H-S287X-FLAG (Fig. 3g, arrows). WiDr cells were additionally transfected with FAM83H-F251/274A-FLAG, which has mutations in the CK1-binding motifs, and, thus, cannot bind to CK1^26^. As expected, FAM83H-F251/274A-FLAG did not induce the localization of CK1α to nuclear speckles, and FAM83H-F251/274A-FLAG itself was also not localized to nuclear speckles (Fig. 3h), suggesting that FAM83H also requires binding to CK1 in order to be localized to nuclear speckles. These results imply that CK1α and FAM83H make a complex and subsequently translocate to nuclear speckles.

### The interactome analysis identified SON as a FAM83H-interacting protein

In HCT116 cells, the N- and C-terminal regions of FAM83H bound to CK1α and keratins, respectively, resulting in the recruitment of CK1α to keratin filaments^8^. We hypothesized that, in RKO cells, the C-terminal region of FAM83H alternatively binds to a protein(s) present in nuclear speckles, resulting in the recruitment of CK1α to nuclear speckles. In order to investigate this hypothesis, we performed an interactome analysis to identify the proteins that bind to the full-length, but not to the N-terminal fragment of FAM83H, namely, those that bind to the C-terminal region of FAM83H. RKO cells were transfected with the vector encoding FAM83H-FLAG, FAM83H-S287X-FLAG, or no insert, and immunoprecipitation using an anti-FLAG antibody was then performed. Co-immunoprecipitated proteins were digested by an in-gel digestion protocol and analyzed using LC-MS/MS. The proteins identified were then ranked by calculating the number of assigned spectra (Table S1). Based on these ranks, SON, protein unc-45 homolog A, and hnRNP M were selected as potential candidates that bind to the C-terminal region of FAM83H in nuclear speckles. Among these three proteins, SON and hnRNP M have been shown to play a role in RNA processing^28^,^29^,^30^,^31^,^32^,^33^; therefore, SON and hnRNP M were selected as more preferential candidates.

**Figure 4:**
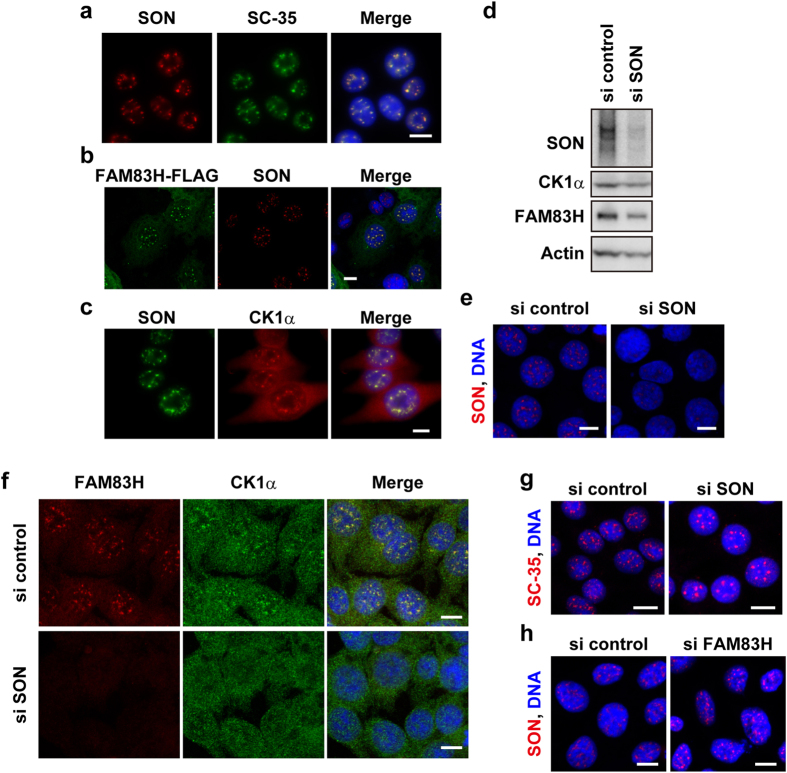
CK1α and FAM83H require SON to be localized to nuclear speckles. (**a–c,e–h**) Immunofluorescence analyses of RKO cells that were transfected with none (**a,c**), a plasmid encoding FAM83H-FLAG (**b**) or the indicated siRNAs (**e–h**). Cells were stained with the indicated antibodies and DAPI (for DNA, blue). Scale bars, 10 μm. (**d**) A Western blot analysis of cells transfected with siRNA for SON or control siRNA.

We investigated whether SON and hnRNP M are localized to nuclear speckles in RKO cells using an immunofluorescence analysis. SON was clearly co-localized with SC-35 (Fig. 4a), as previously shown in HeLa cells^29^,^34^,^35^, and additionally with FAM83H-FLAG and CK1α (Fig. 4b,c); however, co-localization between SON and endogenous FAM83H was not tested due to technical issues. On the other hand, hnRNP M was not detected in nuclear speckles (data not shown). Thus, we hereafter examined the involvement of SON in the localization of CK1α and FAM83H to nuclear speckles.

### SON is required for the localization of CK1α and FAM83H to nuclear speckles

We examined whether the knockdown of SON prevented CK1α and FAM83H from being localized to nuclear speckles. The efficient depletion of SON by RNAi was confirmed by Western blotting and immunofluorescence analyses (Fig. 4d,e). The localization of FAM83H and CK1α, but not SC-35 to nuclear speckles disappeared in SON-depleted cells (Fig. 4f,g), although the staining intensity of FAM83H appeared to be low in SON-depleted cells for an as yet unknown reason (Fig. 4f). On the other hand, the localization of SON to nuclear speckles was not prevented by the knockdown of FAM83H (Fig. 4h). These results suggest that SON is a scaffold protein that directly or indirectly anchors CK1α and FAM83H to nuclear speckles.

### The localization of CK1δ and ε to nuclear speckles is also regulated by FAM83H

**Figure 5:**
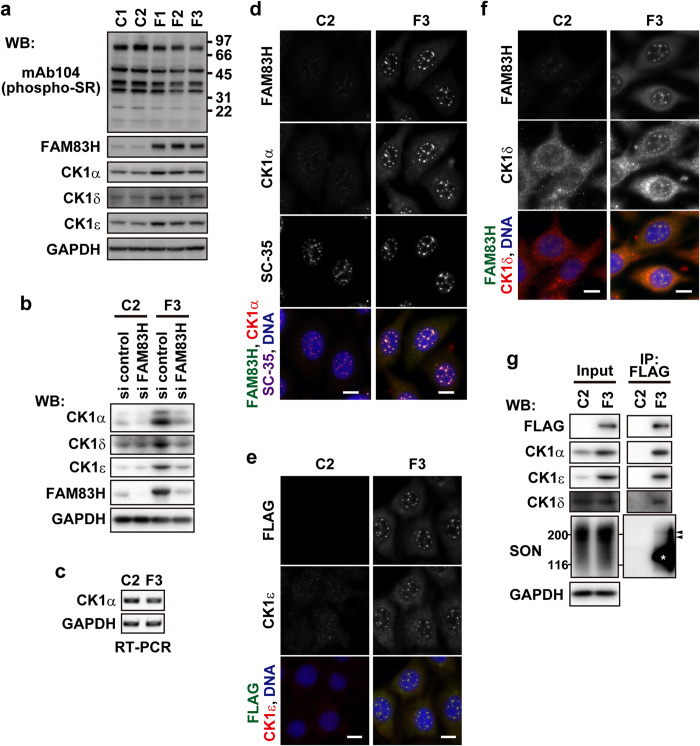
The expression and subcellular localization of CK1α, δ, and ε are regulated by FAM83H. (**a**) RKO cells that are stably transfected with the FAM83H-FLAG vector (F1, 2, and 3) or the empty vector (C1 and 2) were analyzed by Western blotting. The numbers at the right side of the top panel indicate the positions of molecular weight markers (kDa). (**b**) RKO-C1 and F3 cells were transfected with siRNA for FAM83H or control siRNA and then analyzed by Western blotting. (**c**) RKO-C1 and F3 cells were analyzed for the expression of the indicated mRNA by RT-PCR. (**d–f**) RKO-C1 and F3 cells were stained with the indicated antibodies and DAPI (for DNA, blue). Scale bars, 10 μm. (**g**) Immunoprecipitates using an anti-FLAG antibody were prepared from RKO-C1 and F3 cells. Input lysates and immunoprecipitates were analyzed by Western blotting. Arrowheads indicate the migrating positions of major fractions of SON. An asterisk indicates the migrating position of FAM83H-FLAG.

We generated RKO cells stably expressing FAM83H-FLAG (RKO-F1, 2, and 3) at several-fold higher levels than those of endogenous FAM83H expressed in control cell lines that were stably transfected with the empty vector (RKO-C1 and 2) (Fig. 5a). We analyzed these cell lines by Western blotting using antibodies against CK1 members and obtained results showing that the expression levels of CK1α, δ, and ε were higher in RKO-F1, 2, and 3 cells than in RKO-C1 and 2 cells (Fig. 5a). The knockdown of FAM83H-FLAG by RNAi reversed the increased expression of these CK1 members in RKO-F3 cells (Fig. 5b). These results suggest that the expression levels of these CK1 members are controlled by FAM83H. An RT-PCR analysis showed that the levels of mRNA, at least of CK1α, were not elevated in RKO-F3 cells (Fig. 5c); thus, the regulation of CK1 expression by FAM83H appears to occur at the post-transcriptional level.

We used RKO-F3 cells to clarify whether FAM83H localizes CK1δ and ε besides CK1α to nuclear speckles. Similar to transiently transfected FAM83H-FLAG, stably transfected FAM83H-FLAG promoted the localization of CK1α to nuclear speckles (Fig. 5d). Although CK1δ and ε were only slightly detected in nuclear speckles in control cells (RKO-C2), their localization to nuclear speckles was strongly promoted in RKO-F3 cells (Fig. 5e,f). We also confirmed the binding of these CK1 members to FAM83H-FLAG in RKO-F3 cells in co-immunoprecipitation assays using an anti-FLAG antibody (Fig. 5g). These results indicate that CK1δ and ε as well as CK1α are localized to nuclear speckles in a FAM83H-dependent manner.

Although the binding of SON to FAM83H-FLAG was also tested, as shown in Fig. 5g, we were unable to reach a concrete conclusion. SON was detected in the immunoprecipitates of FAM83H-FLAG (Fig. 5g, arrowheads); however, the interpretation of the results obtained was complicated by a non-specific reaction of the primary or secondary antibody possibly against FAM83H-FLAG (Fig. 5g, the asterisk).

### CK1 and FAM83H are not responsible for the phosphorylation of SR proteins

Gross *et al*. previously showed using an mAb104 antibody, which recognizes phospho-SR proteins^36^, that CK1α phosphorylates SR proteins *in vitro*^3^. In order to examine whether the phosphorylation of SR proteins is promoted by the accumulation of CK1 members in nuclear speckles, we performed a Western blot analysis of RKO-F1, 2, and 3 cells, in which CK1α, δ, and ε strongly accumulated in nuclear speckles, as already shown in Fig. 5d–f, using the mAb104 antibody. The immunoreaction of the mAb104 antibody was not stronger in RKO-F1, 2, or 3 cells than in RKO-C1 and 2 cells (Fig. 5a); thus, these results imply that SR proteins are not suitable substrates for CK1 members in RKO cells.

### CK1 and FAM83H do not mediate SON-dependent RNA processing

**Figure 6:**
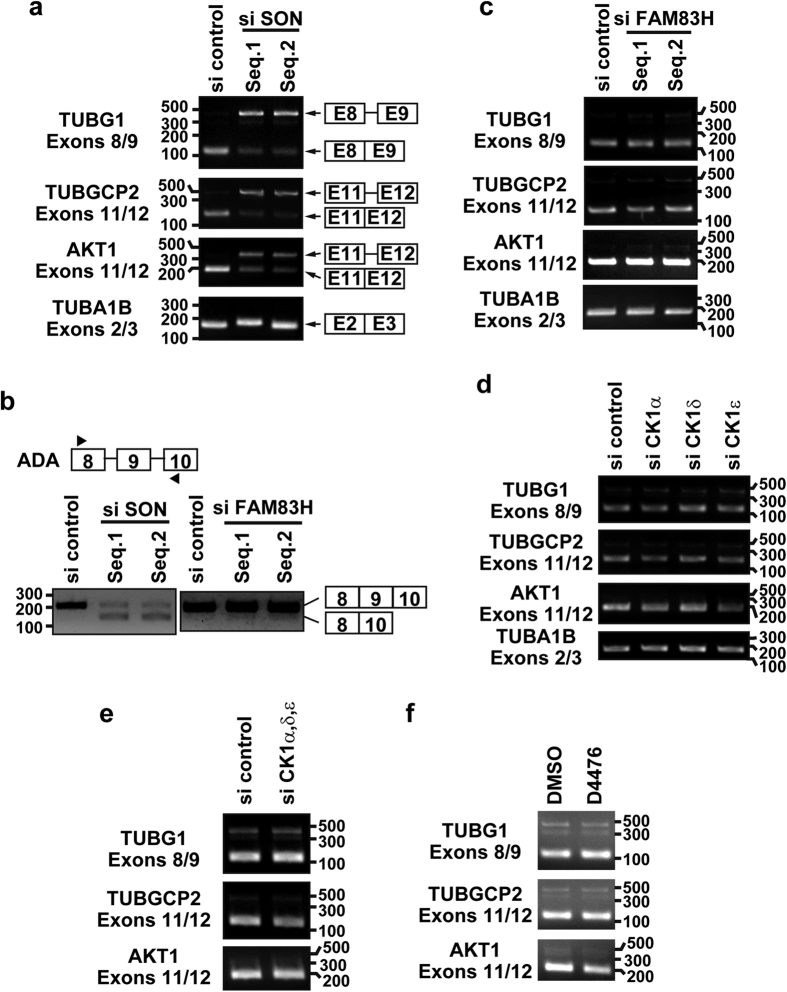
CK1 and FAM83H are not involved in SON-dependent mRNA processing. RKO cells were transfected or treated with siRNA for SON (**a,b**) FAM83H (**b,c**) each or all of CK1α, δ, and ε (**d,e**) or 100 μM D4476 (**f**). Inhibition of the splicing of the indicated introns (**a,c–f**) or induction of the skipping of ADA exon 9 (**b**) was assessed by RT-PCR. The numbers at the left or right of panels indicate the migrating positions of DNA markers (base pairs).

Ahn *et al*. showed that SON is required for the efficient removal of introns at constitutive splice sites on a selective group of genes^29^. Sharma *et al*. also demonstrated that SON maintains accurate splicing for a subset of mRNAs^28^. We investigated whether CK1 and FAM83H mediate mRNA processing in RKO cells. As reported previously by Ahn *et al*., the knockdown of SON inhibited the removal of introns on the mRNAs of TUBG1 (exons 8/9), TUBGCP2 (exons 11/12), and AKT1 (exons 11/12), but not that of the intron between exons 2 and 3 on TUBA1B (Fig. 6a). Furthermore, as previously shown by Sharma *et al*., the skipping of exon 9 of ADA was caused by the depletion of SON (Fig. 6b). This mRNA processing was not markedly affected by the knockdown of FAM83H (Fig. 6b,c), the knockdown of each or all of CK1α, δ, and ε (Fig. 6d,e), or a treatment with the CK1 inhibitor, D4476 (Fig. 6f). These results suggest that CK1 and FAM83H are not involved in SON-dependent splicing processes. Further studies are needed in order to clarify the function of CK1 and FAM83H in nuclear speckles in colorectal cancer cells.

## Discussion

Although CK1α is known to be localized to nuclear speckles, the underlying mechanisms have not yet been elucidated. In the present study, we demonstrated that CK1α requires FAM83H and SON to be localized to nuclear speckles. Our results suggest that CK1α is recruited to nuclear speckles by FAM83H-mediated anchoring to SON.

We previously demonstrated that CK1α is localized on keratin filaments in HCT116 and DLD1 cells^8^. In these cells, FAM83H recruits CK1α to keratin filaments^8^. In the present study, we showed that CK1α is localized to nuclear speckles in RKO cells and WiDr cells in a FAM83H-dependent manner. In the latter cell group, FAM83H has been suggested to directly or indirectly recruit CK1α to SON, which is present in nuclear speckles. Our results indicate that these cell type-specific differences in the subcellular localization of CK1α depend on the presence or absence of an intact keratin cytoskeleton. HCT116 and DLD1 cells expressed simple epithelial keratins 8, 18, and 19, whereas RKO and WiDr cells did not (Fig. 2e). Additionally, specific interactions between keratin proteins and FAM83H were detected in HCT116 cells^8^, but not in RKO cells, as assessed by an interactome analysis of FAM83H-FLAG (Table S1). FAM83H and CK1α translocated into nuclear speckles from keratin filaments in DLD1 cells when rigid and long keratin filaments were largely disrupted by the transfection with siRNAs for type I keratins 18 and 19 (Fig. 2f). These results suggest that FAM83H recruits CK1α to SON in the absence of an intact keratin cytoskeleton, which serves as a more suitable scaffold for FAM83H and CK1α than SON. The localization of CK1α to nuclear speckles has also been reported in NRK, NIH3T3, and Cos-7 cells^3^,^20^. NIH3T3 and Cos-7 cells are fibroblastic cell lines, and NRK cells are a mixture of fibroblastic and epithelial cells^37^. Since keratin proteins are typically expressed in epithelial cells^38^, we did not expect these fibroblastic cell lines to express keratin proteins.

Our results suggest that the protein level of CK1 is also regulated by FAM83H. In RKO cells stably expressing FAM83H-FLAG, we observed increases in the protein levels of CK1α, δ, and ε, but not in their mRNA levels, at least those of CK1α (Fig. 5), suggesting that the increased expression of CK1 is mediated by a post-transcriptional mechanism. In contrast to FAM83H-FLAG, FAM83H-F251/274A-FLAG, which cannot bind to CK1^26^, did not increase the expression of CK1α in WiDr cells, as assessed by immunostaining using an anti-CK1α antibody (Fig. 3f,h, arrows). These results suggest that CK1 is stabilized by binding to FAM83H.

The involvement of FAM83H in the regulation of CK1 expression complicates the interpretation of the phenotype whereby the overexpression of FAM83H promotes the accumulation of CK1 in nuclear speckles. The following two possibilities have been proposed: the overexpression of FAM83H actively promotes the recruitment of CK1 to SON, and the accumulation of CK1 into nuclear speckles is a consequence of the increased expression of CK1 induced by the overexpression of FAM83H. Since the knockdown of type I keratins 18 and 19 in DLD1 cells also induced the translocation of CK1α together with FAM83H into nuclear speckles (Fig. 2f), it is not conceivable that the localization of CK1 to nuclear speckles depends only on its expression level.

Our results further revealed that the subcellular localization and expression of CK1δ and ε are also regulated by FAM83H. CK1δ and ε as well as CK1α bound to FAM83H (Fig. 5g). Our proteomic analysis suggested that CK1α, δ, and ε all bind to the N-terminal region of FAM83H (Table S1). We previously revealed that CK1α binds to the F-X-X-X-F motifs (X, any amino acid residue) in the N-terminal region of FAM83H (FMWSF, 247-251 A.A.; FDEEFRILF, 270-278 A.A.)^8^,^26^. An F-X-X-X-F motif has been shown to serve as a common binding site, at least for CK1α and ε^39^. These results indicate that the subcellular localization and expression of these CK1 members are regulated by similar mechanisms that are dependent on binding to FAM83H.

Although CK1α has been suggested to play a role in mRNA processing^3^,^21^, we were unable to detect the involvement of CK1 or FAM83H. Gross *et al*. showed that CK1α phosphorylates SR proteins *in vitro*^3^. However, our results suggest that CK1α does not phosphorylate SR proteins in RKO cells. As assessed by Western blotting using the mAb104 antibody, we did not observe an increase in the phosphorylation of SR proteins in RKO cells stably expressing FAM83H-FLAG, in which CK1α strongly accumulated in nuclear speckles (Fig. 5). Additionally, although SON is known to play an important role in some types of mRNA processing^28^,^29^,^30^, we were unable to detect the involvement of CK1 and FAM83H in SON-dependent mRNA processing (Fig. 6). We intend to perform a transcriptomic analysis of RKO cells showing the altered expression of CK1 or FAM83H in future studies in order to clarify whether CK1 and FAM83H play a role in mRNA processing.

We observed the localization of FAM83H to nuclear speckles in a small subset of colorectal cancer tissues (Fig. 1), but were unable to establish whether CK1 was co-localized with FAM83H in nuclear speckles in colorectal cancer tissues. It is important to note that we found *in vivo* that the localization of FAM83H to nuclear speckles occurs in colorectal cancer cells with a poorly organized keratin cytoskeleton. Although vimentin, an EMT marker, was not detected in cancer cells showing the nuclear speckle localization of FAM83H in the colorectal cancer tissue tested in Fig. 1d, we speculate that EMT may be one of the events localizing FAM83H to nuclear speckles in other colorectal cancer tissues. The localization of FAM83H to nuclear speckles was observed in cancer cells positioned at the edge of and often detached from the tumor mass. This result implies that FAM83H and CK1 in nuclear speckles play a role in the invasion of colorectal cancer cells.

## Experimental Procedures

### Plasmids and siRNAs

Plasmids encoding FAM83H-FLAG, FAM83H-S287X-FLAG, FAM83H-F251/274A-FLAG, or no insert were generated in previous studies^8^,^26^. siRNAs for FAM83H (FAM83H-HSS138852 and FAM83H-HSS138851) were purchased from Thermo Fisher Scientific (Waltham, MA, USA). siRNAs for SON (Hs_SON_2940 and Hs_SON_9488), CK1α (Hs_CSNK1A_5646), CK1δ (Hs_CSNK1D_1883), CK1ε (Hs_CSNK1E_7959), keratin 18 (Hs_KRT18_2122), and keratin 19 (Hs_KRT19_5383) were purchased from Sigma-Aldrich (St. Louis, MO, USA). Control siRNAs were purchased from Thermo Fisher Scientific (Medium GC Duplex #2) and Sigma-Aldrich (Mission_SIC_001).

### Cell culture and transfection

HCT116, RKO, WiDr, and Caco-2 colorectal cancer cells and an MAb104 hybridoma were purchased from ATCC (Manassas, VA, USA). DLD1 cells were kindly provided by Dr. Tagawa (Chiba Cancer Center Research Institute, Chiba, Japan). These cell lines, except for the MAb104 hybridoma, were maintained at 37 °C in 5% CO_2_ in IMDM (Life Technologies, Carlsbad, CA, USA) or DMEM (Nissui Pharmaceutical, Tokyo, Japan) supplemented with 5 or 10% FBS. The MAb104 hybridoma was cultured in GIT medium (Nihon Pharmaceutical Co., LTD., Tokyo, Japan). Plasmid DNAs and siRNAs were transfected using Lipofectamine 2000 and Lipofectamine RNAiMAX, respectively (Life Technologies). Regarding stable transfection with a plasmid, cells were selected with 800 μg/mL G418 (Nacalai Tesque, Kyoto, Japan). The treatment of cells with D4476 was performed at a concentration of 100 μM for 3 h (Abcam, Cambridge, UK).

### Antibodies

The following antibodies were purchased: anti-FAM83H (HPA024604; Sigma-Aldrich), anti-keratin 8 (TS1; Thermo Fisher Scientific), anti-keratin 18 (DC10; Thermo Fisher Scientific), anti-keratin 19 (RCK108; Thermo Fisher Scientific), anti-CK1α (C-19; Santa Cruz Biotechnology, Santa Cruz, CA, USA), anti-CK1ε (HPA026288; Sigma-Aldrich), anti-CK1δ (R-19; Santa Cruz Biotechnology), anti-SON (HPA023535; Sigma-Aldrich), anti-actin (C-11; Santa Cruz Biotechnology), anti-SC-35 (S4045; Sigma-Aldrich), anti-FLAG (M2; Sigma-Aldrich), anti-hnRNP M (GTX114999; GeneTex, San Antonio, TX, USA), and anti-GAPDH (GT239; GeneTex) antibodies. We used MAb104 hybridoma culture fluid to detect phospho-SR proteins. Alexa Fluor 488, 594, and 647 donkey anti-mouse IgG, Alexa Fluor 488, 555, and 594 donkey anti-rabbit IgG, and Alexa Fluor 488, 555, and 647 donkey anti-goat IgG antibodies were used for immunofluorescence (Life Technologies). HRP-conjugated horse anti-mouse IgG (Cell Signaling Technology, Beverly, MA, USA), donkey anti-rabbit IgG (GE Healthcare, Little Chalfont, UK), bovine anti-goat IgG (Jackson ImmunoResearch, West Grove, PA, USA), and donkey anti-mouse IgM (Jackson ImmunoResearch) antibodies were used for Western blotting.

### Tissue samples from colorectal cancer patients

Colorectal cancer tissues were collected in the Department of Frontier Surgery, Chiba University Hospital, as previously described^8^. Paraffin-embedded blocks were cut into 2.5-μm-thick sections using the microtome, REM-710 (Yamato, Saitama, Japan). Immunostaining was performed with the diaminobenzidine (DAB) chromogen (EnVision + Kit/HRP; Dako, Glostrup, Denmark) and nuclei were stained with Mayer’s hematoxylin (Muto Pure Chemicals, Tokyo, Japan). Stained sections were scanned on a NanoZoomer RS digital slide imaging system (Hamamatsu Photonics, Hamamatsu, Japan). Tissues used for immunofluorescence were embedded in OCT compound (Sakura Finetek, Tokyo, Japan), snap-frozen in liquid nitrogen, and cut into 5-μm-thick sections using a cryostat (Hyrax C50; Carl Zeiss, Jena, Germany). The procedure for the immunofluorescence of tissues was described below. The protocol for the collection and use of tissue samples was approved by the Ethics Committees of the Graduate School of Medicine, Chiba University and the Proteome Research Center, National Institutes of Biomedical Innovation, Health, and Nutrition. Written informed consent was obtained from each patient before surgery.

### Protein extraction, immunoprecipitation, and Western blotting

Protein extraction, immunoprecipitation, and Western blotting were performed as previously described^8^. In the Western blot analysis, cells were directly lysed in SDS-PAGE sample buffer. Regarding immunoprecipitation (IP lysates), cells were suspended in PBS containing 1% NP40 (Fig. 3d, Table S1) or Triton X-100 (Fig. 5g), complete protease inhibitor cocktail (Roche, Basel, Switzerland), and PhosSTOP phosphatase inhibitor cocktail (Roche), and then homogenized by sonication (BIORUPTOR, COSMO BIO, Tokyo, Japan). Immunoprecipitation was performed using the anti-FLAG antibody covalently cross-linked (Fig. 3d, Table S1) or not cross-linked (Fig. 5g) to Protein G Dynabeads (Life Technologies) by dimethyl pimelimidate dihydrochloride (Nacalai Tesque). Western blotting was performed using the chemiluminescence detection system ECL and ECL Prime (GE Healthcare, Little Chalfont, UK) or ChemiLumi One L, Super, and Ultra (Nacalai Tesque). Images were obtained with LAS4000 (Fuji Film, Tokyo, Japan) or ChemiDoc (Bio-Rad, Hercules, CA, USA) and processed with Photoshop CS5 (Adobe, San Jose, CA, USA).

### Reverse transcription (RT)-PCR

The extraction of total RNA was performed using a QIA shredder and RNeasy Mini Kit (Qiagen; Venlo, Netherlands). Reverse transcription was performed using a First Strand cDNA Synthesis Kit for RT-PCR (AMV) (Roche). PCR reactions were performed using the following primer sets: TUBG1, 5′-CGGCTACACCCCTCTCACTA-3′ and 5′-CTGTGGACACCATCACGTTC-3′; TUBGCP2, 5′-GG**A**GGCCTTCTCTTTCGACT-3′ and 5′-TTTTGTTGCTGATCCAGACG-3′; AKT1, 5′-ACAAGGACGGGCACATTAAG-3′ and 5′-ACCGCACATCATCTCGTACA-3′; TUBA1B, 5′-CCGGGCTGTGTTTGTAGACT-3′ and 5′-GATCTCCTTGCCAATGGTGT-3′; ADA, 5′-GCTACCACACCCTGGAAGAC-3′ and 5′-GGGTGGACTTGAAGATGAGC-3′; CK1α, 5′- CTTGGTATTGAGCAGAGTCG-3′ and 5′- TGGTTCAGGGTCCTGAAAAG-3′.

### Immunofluorescence

Immunofluorescence was performed as previously described^8^,^40^. Briefly, cultured cells or tissue sections were fixed with MeOH at −20 °C for 2 min, blocked on ice using Blocking One (Nacalai Tesque), and then sequentially incubated with appropriate primary and secondary antibodies at room temperature. DNA was stained with 100 ng/mL 4′-6-diamidino-2-phenylindole (DAPI; Sigma-Aldrich), and stained samples were viewed under an IX83 fluorescence microscope with UPlan FL N 40x/0.75 and PlanApo N 60x/1.42 objective lenses (Olympus, Tokyo, Japan) or an LSM710 confocal microscope with EC Plan-NEO FLUAR 40x/1.3 and Plan APOCHROMAT 63x/1.4 objective lenses (Carl Zeiss, Jena, Germany). Composite figures were prepared using Photoshop CS5.

### Protein identification by LC-MS/MS

Protein identification by LC-MS/MS was performed as previously described^8^. In brief, immunoprecipitates with an anti-FLAG antibody were resolved by SDS-PAGE and the gel lane was divided into 7 pieces corresponding to different molecular masses. The in-gel tryptic digestion of proteins was performed as previously described^41^. The digested peptides were analyzed using an LTQ-Orbitrap Velos mass spectrometer (Thermo Scientific). A 0.3 × 5 mm trap column (L-column ODS; Chemicals Evaluation and Research Institute (CERI), Tokyo, Japan) and an analytical column made in-house by packing L-column2 C18 (CERI) into a self-pulled needle (0.1 × 200 mm) were used^42^. The mobile phases consisted of buffers A (0.1% formic acid and 2% acetonitrile) and B (0.1% formic acid and 90% acetonitrile). The nanoLC gradient was delivered at 500 nL/min and consisted of a linear gradient of buffer B developed from 5 to 35% B in 45 min. The dynamic exclusion function of LTQ-Orbitrap was turned off. Regarding protein identification, peptide mass data were matched by searching the UniProtKB/Swiss-Prot database (2011_12) using the MASCOT search engine v2.3. Database search parameters were: the charge of the precursor ion, 2+ and 3+; peptide mass tolerance, 3 ppm; fragment tolerance, 0.6 Da; allowing up to one missed cleavage; fixed modification, the carbamidomethylation of cysteine; variable modification, the oxidation of methionine. Proteins were identified based on at least 2 unique peptides. The number of assigned spectra was calculated using Scaffold 3 software (Proteome Software, Portland, OR, USA) for semi-quantitation.

## Additional Information

**How to cite this article**: Kuga, T. *et al*. Casein kinase 1 is recruited to nuclear speckles by FAM83H and SON. *Sci. Rep.* **6**, 34472; doi: 10.1038/srep34472 (2016).

## Supplementary Material

Supplementary Information

Supplementary Table S1
