# Supplementary material for: Casein kinase 1 is recruited to nuclear speckles by FAM83H and SON
Source: Sci Rep. 2016 Sep 29;6:34472. doi: 10.1038/srep34472 (PMC5041083; doi:10.1038/srep34472)
Supplement: Supplementary Information [file srep34472-s1.doc]

**Casein kinase 1 is recruited to nuclear speckles by FAM83H and SON**

**Takahisa Kuga, Hideaki Kume, Jun Adachi, Naoko Kawasaki,**

**Maiko Shimizu, Isamu Hoshino, Hisahiro Matsubara, Youhei Saito,**

**Yuji Nakayama, and Takeshi Tomonaga**

**Supplemental Table**

**Table S1 Identification of co-immunoprecipitated proteins with FAM83H-FLAG and FAM83H-S287X-FLAG.**

Immunoprecipitates with an anti-FLAG antibody from RKO cells transiently transfected with plasmids encoding FAM83H-FLAG, FAM83H-S287X-FLAG, or no insert (control) were analyzed by LC-MS/MS. The identified proteins and numbers of assigned spectra are shown. The ratios of assigned spectral numbers for each protein between individual samples were calculated. Asterisks (*), not identified in the reference immunoprecipitates. Total/kDa, the total number of assigned spectra for each protein was normalized by the molecular weight.
